# Instructed learning strategy use eliminates negative reactivity of immediate judgments of learning

**DOI:** 10.3758/s13423-025-02844-w

**Published:** 2026-01-14

**Authors:** Franziska Ingendahl, Monika Undorf

**Affiliations:** Department of Psychology, Technical University of Darmstadt, Alexanderstr. 10, 64283 Darmstadt, Germany

**Keywords:** Metamemory, Judgments of learning, Reactivity, Learning strategies

## Abstract

Predicting one’s own future memory during learning (immediate judgments of learning, JOLs) can reactively alter memory performance. Recent evidence shows that making JOLs is associated with changes in the spontaneous use of learning strategies and that these changes mediate negative effects of immediate JOLs on cued recall of unrelated word pairs. This study tests whether a learning strategy instruction targeted at JOL-induced changes in spontaneous learning strategy use reduces negative JOL reactivity. Two experiments (Experiment 1: *N* = 193, Experiment 2: *N* = 200) compared cued recall of related and unrelated word pairs between groups of participants who (a) provided JOLs during study, (b) provided JOLs during study and were instructed to study unrelated pairs using mental imagery (Experiment 1) or any learning strategy (Experiment 2), or (c) did not provide JOLs and did not receive a learning strategy instruction. In both experiments, making JOLs without learning strategy instructions impaired memory performance for unrelated word pairs compared to not making JOLs (negative JOL reactivity). Importantly, learning strategy instructions eliminated negative JOL reactivity. Together with findings on spontaneous learning strategy use, these results indicate that negative JOL reactivity may be due to changes in learning strategy use, aligning with theoretical accounts that attribute negative JOL reactivity to dual-task costs or changes in goals pursued during learning.

**Supplementary Information:**

The online version contains supplementary material available at 10.3758/s13423-025-02844-w.

For a long time, metacognition researchers assumed that asking participants to predict their memory performance immediately after learning (immediate judgments of learning, JOLs; Rhodes, 2016) would not alter learning and memory (Jordano & Touron, 2018). However, recent research shows that soliciting immediate JOLs can alter learning and memory—a phenomenon called *JOL reactivity* (for a meta-analysis, see Ingendahl et al., 2025).

In a typical experiment examining reactive effects of immediate JOLs, researchers ask two groups of participants to study word pairs for a memory test (e.g., Soderstrom et al., 2015). While one group studies each pair for a fixed duration without providing JOLs (*no-JOL group*), the other group studies each pair and provides a JOL within the same duration (*JOL group*). Using this paradigm, researchers have obtained three types of reactivity—positive reactivity, negative reactivity, and no reactivity. *Positive reactivity* refers to better memory performance in the JOL group than in the no-JOL group and is often found for related pairs (e.g., Janes et al., 2018; Rivers et al., 2023; Soderstrom et al., 2015), backward related pairs (e.g., Maxwell & Huff, 2022), and identical pairs (e.g., Halamish & Undorf, 2023) tested in cued-recall (e.g., Rivers et al., 2023; Soderstrom et al., 2015; Witherby & Tauber, 2017), and recognition tests (e.g., Maxwell & Huff, 2024; Myers et al., 2020). *Negative reactivity* refers to worse memory performance in the JOL group than in the no-JOL group and has been observed for unrelated pairs in cued-recall tests (e.g., Ingendahl & Undorf, 2025; Mitchum et al., 2016; Undorf et al., 2024). *No reactivity* refers to similar memory performance in JOL and no-JOL groups and has been found for unrelated pairs (e.g., Myers et al., 2020; Soderstrom et al., 2015) and various other item types in free-recall tests (e.g., Myers et al., 2020; Zhao et al., 2023).

Much research has focused on understanding mechanisms driving positive reactivity for related pairs (e.g., Chang & Brainerd, 2022; Halamish & Undorf, 2023; Maxwell & Huff, 2022; Rivers et al., 2021). In contrast, much less is known about mechanisms driving negative reactivity for unrelated pairs (but see Janes et al., 2018; Mitchum et al., 2016). One reason may be that published studies more often report positive reactivity for related pairs than negative reactivity for unrelated pairs. A recent meta-analysis (Ingendahl et al., 2025) indicates that this imbalance reflects both smaller effects sizes for negative reactivity and a publication bias against findings of negative reactivity. Given that negative reactivity is a robust phenomenon (Ingendahl et al., 2025; Undorf et al., 2024), clarifying its underlying mechanisms is essential.

A recent study by Ingendahl and Undorf (2025) examined the role of spontaneous learning strategies for JOL reactivity (but see Mitchum et al., 2016; Rivers et al., 2021).^1^ It showed that the frequency of learning strategy use was not reliably associated with positive reactivity for related pairs, but consistently contributed to negative reactivity for unrelated pairs. Across multiple experiments, JOL-group participants reported using mental imagery less frequently and no learning strategy more frequently for studying unrelated pairs than no-JOL group participants, and these differences mediated negative reactivity for unrelated pairs. Moreover, Double and colleagues (2025b) showed that learning strategy instructions can mitigate negative JOL reactivity. Specifically, providing hints about the relational rule predicting category membership eliminated negative JOL reactivity in rule-based category learning (also see Double et al., 2025a).

Links between negative JOL reactivity and learning strategy use align with two prominent theoretical accounts explaining negative JOL reactivity. The *dual-task account* (Mitchum et al., 2016) assumes that negative reactivity occurs because making JOLs during learning requires resources and interferes with the primary task of studying, specifically when it is difficult (e.g., when studying unrelated pairs). This interference may impede learning strategy use. The *changed-goal account* (Mitchum et al., 2016) proposes that soliciting JOLs shifts people’s learning goal from mastering all pairs to focusing on pairs perceived as easier (e.g., related pairs) and neglecting pairs perceived as more difficult (e.g., unrelated pairs). Neglecting unrelated pairs may reduce learning strategy use for unrelated pairs.

Previous research on the contribution of learning strategies to negative reactivity in the context of word-pair learning has exclusively relied on assessing spontaneous learning strategy use. It thus provides only correlative evidence, leaving it unclear whether changing learning strategy use would reduce negative reactivity for unrelated pairs.

## Present study

The present study aims to shed further light on the relationship between learning strategies and negative JOL reactivity by manipulating learning strategy use. In two experiments, we test whether instructing a JOL group to use mental imagery (Experiment 1) or any learning strategy (Experiment 2) for studying unrelated pairs reduces negative reactivity for unrelated pairs compared to a standard JOL group without strategy instructions.

While no previous research addressed the effects of instructed use of any learning strategy on JOL reactivity, Witherby and colleagues (2023) examined whether positive reactivity transfers from pre-existing relationships of related pairs to relationships between unrelated words formed by mental imagery. Comparing participants who made or did not make JOLs and received or did not receive mental-imagery instructions, they showed that mental-imagery instructions improved memory but did not affect reactivity: Making JOLs always enhanced memory for related pairs and impaired memory for unrelated pairs. By showing that mental-imagery instructions did not affect negative reactivity when comparing mental-imagery JOL and no-JOL groups, the study provides important insights into the interactions between mental imagery and JOL reactivity. It does not, however, clarify whether mental-imagery instructions enhance memory performance sufficiently to eliminate negative reactivity compared to a standard no-JOL group. Also, Witherby and colleagues (2023) instructed participants to use mental imagery for all pairs. Such global strategy instructions may, however, carry the risk of altering learners’ general study approach. Given evidence that learning strategies specifically contribute to reactivity for unrelated pairs (Ingendahl & Undorf, 2025), global learning strategy instructions may not be suitable for examining the role of learning strategies in negative reactivity.

The present study therefore examines the impact of learning-strategy instructions for only unrelated pairs on negative reactivity for unrelated pairs compared to standard JOL groups. In two experiments, we specifically target learning strategies previously found to mediate negative reactivity for unrelated pairs (see Ingendahl & Undorf, 2025). Experiment 1 examines whether instructions to study unrelated pairs using mental imagery reduce negative JOL reactivity. Experiment 2 tests whether instructions to use any learning strategy (as opposed to no learning strategy) for studying unrelated pairs reduces negative JOL reactivity.

## Method

We report how we determined our sample size, all data exclusions, all manipulations, and all measures. Both experiments follow the Journal Article Reporting Standard (JARS; Appelbaum et al., 2018) and were conducted in line with international and local ethics guidelines; they were exempt from review by the local ethics committee. The materials, data, and analytical code of both experiments are available at osf.io/3pnk9. Both Experiments were preregistered and the preregistrations are available at the Open Science Framework, Experiment 1: osf.io/wg325; Experiment 2: osf.io/y4jek.

### Design and participants

#### Experiment 1

Experiment 1 used a 2 (relatedness: related, unrelated) *×* 3 (judgment group: JOL, Mental-Imagery [MI]-JOL, no JOL) mixed design with relatedness manipulated within participants and judgment group manipulated between participants. An a priori power analysis using G*Power (Faul et al., 2007) indicated that 64 participants per group would be sufficient for detecting medium-sized effects (*d* = 0.50; e.g., see Soderstrom et al., 2015, Experiment 1b; also see Ingendahl et al., 2025) in two-tailed *t* tests for independent samples with $\alpha$ = .05 and (1–$\beta$) = .80. Further, a sensitivity analysis showed that a total sample size of *N* = 192 participants allowed for detecting a small-sized (*f* = .11) interaction between relatedness and judgment group in a mixed ANOVA.^2^

To arrive at the preregistered sample size, we recruited 199 German university students in exchange for partial course credit. We excluded two participants reporting technical malfunctions, two participants reporting deception (such as writing down words during study), and two participants due to not providing JOLs in more than 20% of the study trials. The final sample comprised *N* = 193 participants (*n* = 65 in the JOL group, *n* = 64 in the MI-JOL group, and *n* = 64 in the no-JOL group). Participants (156 women, 37 men, 0 diverse) had a mean age of 22.10 years (*SD* = 3.34) and were randomly assigned to one of the judgment groups.

#### Experiment 2

Experiment 2 used a 2 (relatedness: related, unrelated) *×* 3 (judgment group: JOL, Learning-Strategy [LS]-JOL, no JOL) mixed design with relatedness manipulated within participants and judgment group manipulated between participants. The sample size rationale and the target sample size were the same as in Experiment 1. To arrive at this sample size, we recruited 213 German university students in exchange for partial course credit. We excluded two participants reporting technical malfunctions, one participant reporting deception, six participants not fluent in German, and four participants due to not providing JOLs in more than 20% of the study trials. The final sample included *N* = 200 participants (*n* = 66 in the JOL group, *n* = 67 in the LS-JOL group, and *n* = 67 in the no-JOL group). Participants (148 women, 51 men, 1 diverse) had a mean age of 22.30 years (*SD* = 3.45) and were randomly assigned to one of the judgment groups.

### Materials

#### Experiment 1

Materials in Experiment 1 were 40 German cue-target word pairs taken from Melinger and Weber (2006). Half were related (e.g., “arch–arrow”) with a mean forward associative strength of .53 (*SD* = .15, range: .33–.88) and half were unrelated (e.g., “owl–soup”) with a mean forward associative strength of .00. Related and unrelated word pairs were equal in length (*M* = 5.05, *SD* = 0.66, range: 4.00–6.50), valence (*M* = 0.71, *SD* = 0.84, range: −2.20–2.16), arousal (*M* = 2.39, *SD* = 0.58, range: 1.75–4.22), frequency per million (*M* = 79.73, *SD* = 96.23, range: 3.83–409.25), and concreteness (*M* = 5.77, *SD* = 0.55, range: 3.77–6.65; normed values for valence, arousal, frequency, and concreteness taken from Lahl et al., 2009). Four additional word pairs (two related, two unrelated) served as buffer items at the beginning of the study phase and three additional unrelated word pairs were used for practicing the mental-imagery strategy in the MI-JOL group. Neither the buffer pairs nor the practice pairs were included in any analysis.

#### Experiment 2

Materials in Experiment 2 were two lists of 20 target words with one related cue word and one unrelated cue word each taken from Melinger and Weber (2006). Each participant was presented with all related word pairs from one list and all unrelated word pairs from the other list, with related and unrelated pairs from both lists presented equally often across participants in the JOL, LS-JOL, and no-JOL groups. Each list contained 20 related word pairs (e.g., “arrow–bow”) with a mean forward associative strength of .50 (*SD* = .10, range: .36–.71) and 20 unrelated word pairs (e.g., “frog–bow”) with a mean forward associative strength of .00. Within and across lists, related and unrelated word pairs were equal in length (*M* = 5.63, *SD* = 0.92, range: 4.00–9.00), valence (*M* = 0.58, *SD* = 0.82, range: −2.50–2.11), arousal (*M* = 2.56, *SD* = 0.57, range: 1.61–4.09), frequency per million (*M* = 54.46, *SD* = 67.22, range: 1.49–409.25), and concreteness (*M* = 5.74, *SD* = 0.49, range: 3.77–6.68; normed values for valence, arousal, frequency, and concreteness taken from Lahl et al., 2009). Four additional word pairs (two related, two unrelated) were used as buffer pairs presented at the beginning of the study phase and three additional unrelated word pairs were used for practicing learning strategies in the LS-JOL group. Neither the buffer pairs nor the practice pairs were included in any analysis.

### Procedure

#### Experiment 1

After providing informed consent, all participants were informed that they would be asked to study 44 word pairs for a later memory test in which they would have to recall the second word of each pair when presented with the first word.

Before proceeding to the study phase, participants in the MI-JOL group received mental-imagery instructions to use the learning strategy mental imagery for studying word pairs presented in purple or green font, respectively:

"During the study phase, some word pairs will be presented in purple (green) font color, while other pairs will be presented in green (purple) font color. Please use the learning strategy mental imagery to study the purple (green) pairs. This learning strategy involves forming a detailed mental image including the two words of a pair during studying. For example, for the pair *spoon–sun*, you might imagine a spoon shining brightly because it is lying in the sun. It is up to you how realistic your image will be. For the pair *dog–guitar*, you could very well imagine a dog playing a guitar. Please make sure that you envision the image in as much detail as possible. You can use any learning strategy you like to study the word pairs in green (purple). Please try to memorize all word pairs. In the later memory test, we will test your memory of word pairs in both purple and green color."

Participants in the MI-JOL group then practiced the application of mental imagery for three unrelated word pairs not included in the study list. They saw each word pair for 6 s each and were instructed to form a detailed mental image including the two words of the pair. Immediately after each word pair, participants were asked to provide a detailed description of their mental image.

At study, all groups studied the word pairs for 6 s each. For a random half of the participants in each group, related word pairs were presented in purple font color, and unrelated word pairs were presented in green font color at study. The other half of the participants saw the related and unrelated word pairs in reversed font-color assignments. Participants in the JOL and MI-JOL groups were prompted after 2 s of study time to provide a JOL (“probability of coming up with the second word when presented with the first word at the later test?”) on a horizontal scale ranging from 0 to 100 percent. If participants in the JOL and MI-JOL groups failed to provide a JOL within the remaining 4 s of study time, they were reminded to provide their JOLs within the available time (“Please give your next judgment within the available time”) for 3 s. After studying all word pairs, participants solved visual reasoning items (Chierchia et al., 2019) for 2 min and then completed a self-paced cued-recall test. In the test, participants were presented with the cue word of each studied pair and typed in the respective target word. Word pairs were presented in new random orders at study and test for each participant.

Afterwards, participants indicated their learning strategies for each studied word pair. After reading short descriptions of the assessed learning strategies (see Ingendahl & Undorf, 2025), participants saw all studied word pairs again in a new randomized order and selected the learning strategy they had used for studying the respective word pair (mental imagery, rote repetition, sentence generation, making connections without generating a mental image or sentence, retrieval practice, other learning strategy, or no strategy). When selecting “other learning strategy,” they were required to describe the learning strategy they had used. If participants did not remember which learning strategy they had used or whether they used a learning strategy at all, they were instructed to select the fallback option “I don’t know”. Finally, participants were asked to complete a translated version of the Vividness of Visual Imagery Questionnaire (Marks, 1973; translation based on Beran et al., 2023) to assess aphantasia.^3^

#### Experiment 2

The procedures for the JOL and no-JOL groups in Experiment 2 were identical to those in Experiment 1. The procedure for the LS-JOL group was identical to that for the MI-JOL group in Experiment 1 with the following exceptions.

Instead of receiving an instruction to use mental imagery for learning the purple (green) word pairs, participants were instructed to explicitly use a learning strategy for studying the purple (green) word pairs. Learning strategy instructions read:

"During the study phase, some word pairs will be presented in purple (green) font color, while other pairs will be presented in green (purple) font color. Please use a learning strategy for studying the purple (green) pairs that will help you to remember these word pairs as accurately as possible in the later memory test. Various learning strategies can help you to remember the purple (green) pairs well. Learning strategies that connect the two words in a pair are particularly suitable for learning the pairs in purple (green). You can connect the two words in different ways. For example, you could imagine an image that contains both words (e.g., a dog playing the guitar for the pair *guitar–dog*), or you can form a sentence with both words (e.g., the sentence *The spoon is lying in the sun* for the pair *spoon–sun*). You could also think of other ways to connect the two words of a pair. There are several other learning strategies that can help you remember the word pairs in purple (green) in the later memory test. You may use other learning strategies than those described here to learn the pairs in purple (green). Any learning strategy that works for you personally is valid."

Unlike in Experiment 1, we did not administer the Vividness of Visual Imagery Questionnaire.

#### Analytical strategy

R (Version 4.3.3; R Core Team, 2024) and R Studio (Version 2025.5.1.513; RStudio Team, 2025) were used for data preparation and all analyses reported in this study. We used *afex* (Version 1.3-1; Singmann et al., 2024) for all reported ANOVAs, *effsize* (Version 0.8.1; Torchiano, 2016) for calculating effect sizes for *t* tests, and *bruceR* (Version 2023.9; Bao, 2023) for all multilevel analyses. The α level was set to .05 for all analyses. All analyses were preregistered unless stated otherwise.

Preliminary exploratory analyses (see Supplementary Materials) showed that the assignment of font color to related and unrelated pairs did not interact with judgment group on JOLs, Experiment 1: *F*s $\le$ 2.77, *p*s $\ge$ .098, ${\eta }_{p}^{2}$ s $\le$ .02; Experiment 2: *F*s $\le$ 0.65, *p*s $\ge$ .420, ${\eta }_{p}^{2}$ s $\le$ .01, use of mental imagery (Experiment 1), *F*s $\le$ 1.06, *p*s $\ge$ .349, ${\eta }_{p}^{2}$ s $\le$ .01, or no strategy (Experiment 2), *F*s $\le$ 2.21, *p*s $\ge$ .113, ${\eta }_{p}^{2}$ s $\le$ .02, or cued-recall performance, Experiment 1: *F*s $\le$ 1.37, *p*s $\ge$ .257, ${\eta }_{p}^{2}$ s $\le$ .01; Experiment 2: *F*s $\le$ 0.75, *p*s $\ge$ .474, ${\eta }_{p}^{2}$ s $\le$ .01. Further exploratory analyses for Experiment 2 showed that list assignment did not interact with judgment group on JOLs, *F*s $\le$ 0.94, *p*s $\ge$ .333, ${\eta }_{p}^{2}$ s $\le$ .01, use of no strategy, *F*s $\le$ 2.35, *p*s $\ge$ .099, ${\eta }_{p}^{2}$ s $\le$ .02, or cued-recall performance, *F*s $\le$ 0.48, *p*s $\ge$ .621, ${\eta }_{p}^{2}$ s $\le$ .01. Therefore, data were collapsed across counterbalancing conditions in all analyses reported below.

We analyzed JOLs with 2 (relatedness: related, unrelated) *×* 2 (judgment group: JOL, MI-JOL/LS-JOL) mixed ANOVAs. We analyzed the use of mental imagery in Experiment 1, the use of no strategy in Experiment 2, and cued-recall performance (both experiments) with 2 (relatedness: related, unrelated) *×* 3 (judgment group: JOL, MI-JOL/LS-JOL, no JOL) mixed ANOVAs. We followed up on significant interactions with two-sided two-sample *t* tests comparing differences between judgment groups separately for related and unrelated word pairs. For reasons of conciseness, we focus on reporting the main effects, interactions, and follow-up analyses directly relevant to our research questions. Specifically, we only present results regarding reported learning strategies for mental imagery in Experiment 1 and for no strategy in Experiment 2.

To test whether differences in learning strategy use statistically explained potential differences in cued-recall performance between the JOL and MI-JOL groups (Experiment 1) as well as JOL and LS-JOL groups (Experiment 2), we ran exploratory multilevel moderated mediation models. To account for differences in JOL reactivity and learning strategy use between related and unrelated word pairs, each model included word-pair relatedness as a moderator of the direct effect of judgment group on cued-recall performance and learning strategy use. In a first step, we entered the learning strategy targeted by the learning strategy instruction (mental imagery in Experiment 1, no strategy in Experiment 2) as a mediator. In a second step, we added the other strategy that contributed to negative JOL reactivity in Ingendahl and Undorf (2025) (no strategy in Experiment 1, mental imagery in Experiment 2). We performed each model with the highest converging random effect structure and included dummy-coded variables for judgment group (Level 2; 0 = JOL group, 1 = MI-JOL/LS-JOL group), word-pair relatedness (Level 1; 0 = unrelated, 1 = related), and learning strategy use (Level 1; 0 = learning strategy not reported, 1 = learning strategy reported).

The Supplementary Materials provide additional preregistered and exploratory analyses, including Bonferroni-Holm corrected *p* values for* t* tests and Bayesian reanalyses for the preregistered main analyses.

## Results

### JOLs

JOLs are presented in Table 1.

**Table 1:** Mean JOLs in Experiments 1 and 2

Experiment and item type	JOLs	
	JOL group	MI/LS-JOL group
Experiment 1		
Related pairs	74.14 (16.88)	74.39 (11.63)
Unrelated pairs	22.43 (13.73)	43.56 (16.94)
Experiment 2		
Related pairs	69.69 (15.80)	70.61 (17.23)
Unrelated pairs	26.00 (15.91)	36.23 (15.63)

Values in parentheses represent one standard deviation. MI/LS-JOL group refers to the MI-JOL group in Experiment 1 and the LS-JOL group in Experiment 2

#### Experiment 1

An ANOVA (Table 2a) revealed significant effects of relatedness and judgment group and a significant interaction. The main effects showed that participants provided higher JOLs for related than for unrelated pairs and that the JOL group provided lower JOLs than the MI-JOL group. The interaction revealed JOL differences between groups for unrelated pairs, *t*(127) = 7.79, *p* < .001, *d* = 1.37, but not for related pairs, *t*(127) = 0.10, *p* = .924, *d* = 0.02.

**Table 2:** Results of mixed ANOVAs with the factors relatedness and judgment group for a) JOLs, b) mental imagery/no strategy, and (c) cued-recall performance

Experiment and effect	a) JOLs (JOL and MI/LS-JOL groups)				b) Mental imagery/no strategy					c) Cued-recall performance			
	*F*	*df*	*p*	${\eta }_{p}^{2}$		*F*	*df*	*p*	${\eta }_{p}^{2}$	*F*	*df*	*p*	${\eta }_{p}^{2}$
Experiment 1													
Relatedness	619.63	1, 127	<.001	.83		116.67	1, 190	<.001	.38	857.03	1, 190	<.001	.82
Judgment group	27.22	1, 127	<.001	.18		23.03	2, 190	<.001	.20	5.78	2, 190	.004	.06
Relatedness $\times$ Judgment group	39.70	1, 127	<.001	.24		53.67	2, 190	<.001	.36	44.03	2, 190	<.001	.32
Experiment 2													
Relatedness	545.70	1, 131	<.001	.81		54.34	1, 197	<.001	.22	630.11	1, 197	<.001	.76
Judgment group	6.14	1, 131	.014	.05		1.19	2, 197	.308	.01	1.03	2, 197	.358	.01
Relatedness $\times$ Judgment Group	7.77	1, 131	.006	.06		8.04	2, 197	<.001	.08	15.08	2, 197	<.001	.13

Relatedness was manipulated within participants, and judgment group was manipulated between participants. The ANOVAs in a) refer to 2 (relatedness: related, unrelated) $\times$ 2 (judgment group: JOL, MI-JOL/LS-JOL) mixed ANOVAs, and the ANOVAs in b) and c) refer to 2 (relatedness: related, unrelated) $\times$ 3 (judgment group: JOL, MI-JOL/LS-JOL, no JOL) mixed ANOVAs

#### Experiment 2

An ANOVA (Table 2a) revealed significant effects of relatedness and judgment group, and a significant interaction. The main effects showed that participants provided higher JOLs for related than for unrelated pairs and that the JOL group provided lower JOLs than the LS-JOL group. The interaction revealed JOL differences between groups for unrelated pairs, *t*(131) = 3.74, *p* < .001, *d* = 0.65, but not for related pairs, *t*(131) = 0.32, *p* = .750, *d* = 0.06.

### Learning strategy use

**Fig. 1:**
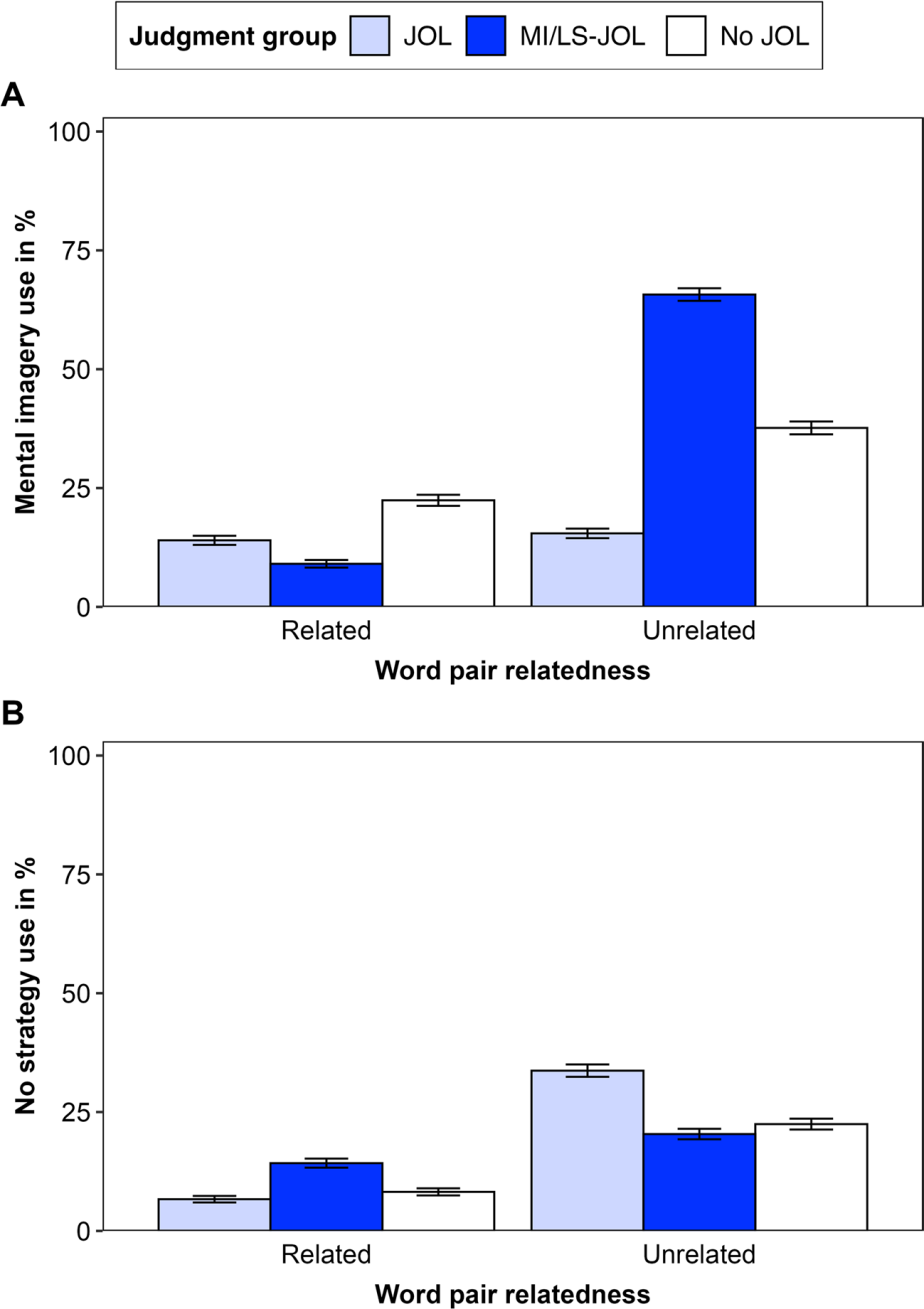
Reported mental imagery use in Experiment 1 (**A**) and no strategy use in Experiment 2 (**B**). *Note.* Error bars represent one standard error of the mean. MI/LS-JOL group refers to the MI-JOL group in Experiment 1 and the LS-JOL group in Experiment 2

Figure 1 presents the frequency of reporting the use of mental imagery in Experiment 1 (Panel A) and no strategy in Experiment 2 (Panel B).

#### Experiment 1

An ANOVA (Table 2b) revealed significant effects of relatedness and judgment group and a significant interaction. The main effects showed that participants used mental imagery more frequently for unrelated than for related pairs and that the JOL group used mental imagery less frequently than the other groups (for follow-up tests, see Supplementary Materials). The interaction showed that the effect of judgment group differed by relatedness. For related pairs, both JOL groups used mental imagery less frequently than the no-JOL group, JOL: *t*(127) = 1.99, *p* = .049, *d* = 0.35; MI-JOL: *t*(126) = 3.38, *p* = .001, *d* = 0.60, and did not differ from each other, *t*(127) = 1.57, *p* = .118, *d* = 0.28. For unrelated pairs, the MI-JOL group used mental imagery more frequently than the no-JOL group, *t*(126) = 5.10, *p* < .001, *d* = 0.90, and the JOL group, *t*(127) = 11.34, *p* < .001, *d* = 2.00, with less frequent strategy use in the JOL than in the no-JOL group, *t*(127) = 4.65, *p* < .001, *d* = 0.82.

#### Experiment 2

An ANOVA (Table 2b) revealed a significant effect of relatedness and a significant interaction. The main effect showed that participants used no strategy more frequently for unrelated than for related pairs. The interaction showed that the effect of judgment group differed by relatedness. For related pairs, there were no group differences, JOL vs no JOL: *t*(131) = 0.52, *p* = .607, *d* = 0.09; JOL vs LS-JOL: *t*(131) = 1.94, *p* = .055, *d* = 0.34; LS-JOL vs no JOL, *t*(132) = 1.59, *p* = .114, *d* = 0.27. For unrelated pairs, the JOL group used no strategy more frequently than the no-JOL group, *t*(131) = 2.37, *p* = .019, *d* = 0.41, and the LS-JOL group, *t*(131) = 2.88, *p* = .005, *d* = 0.50, with no difference between the two latter groups, *t*(132) = 0.48, *p* = .629, *d* = 0.08.

### Cued recall

**Fig. 2:**
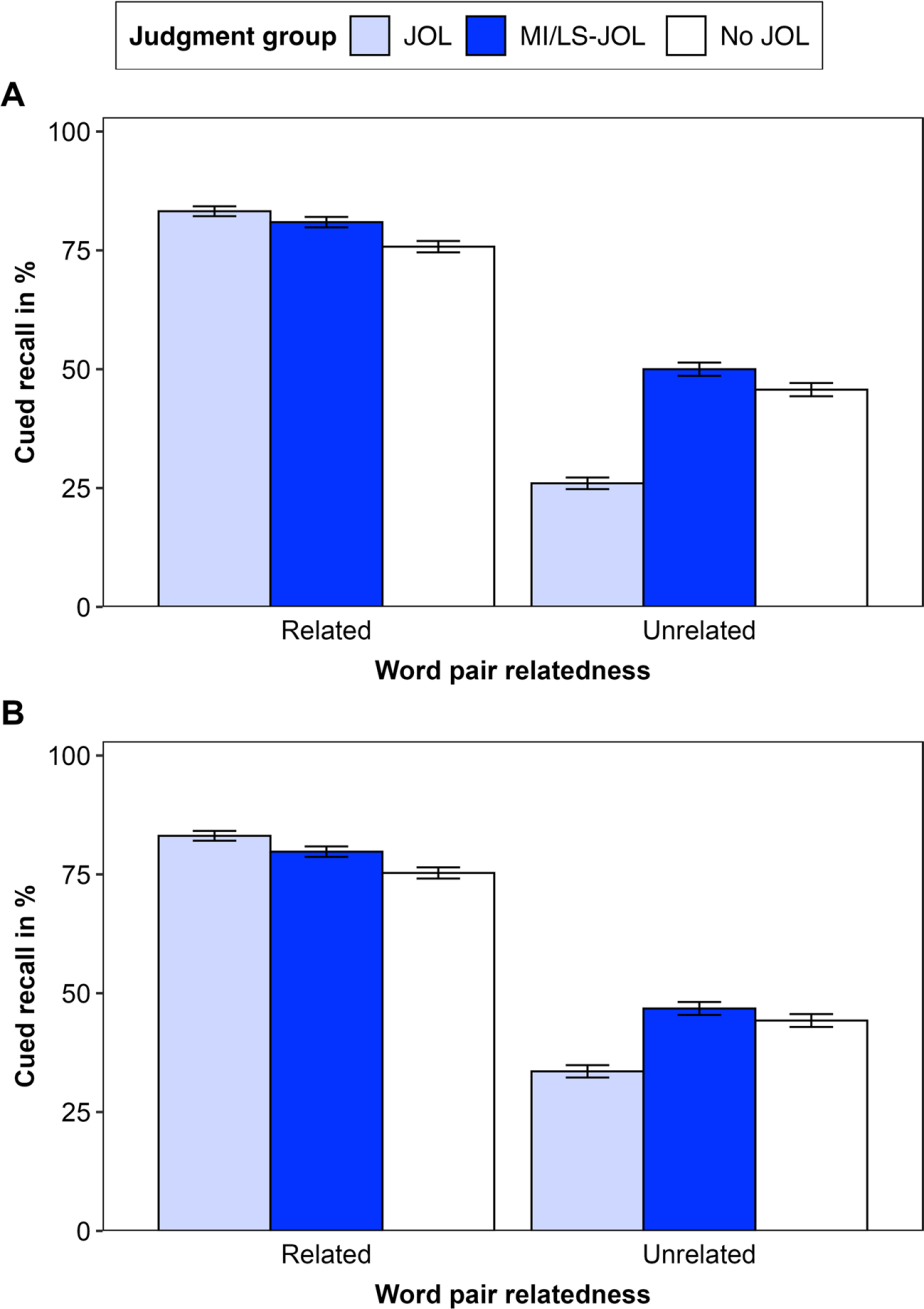
Cued-recall performance in Experiment 1 (**A**) and Experiment 2 (**B**). *Note.* Error bars represent one standard error of the mean. MI/LS-JOL group refers to the MI-JOL group in Experiment 1 and the LS-JOL group in Experiment 2

Figure 2 presents cued-recall performance in Experiments 1 (Panel A) and 2 (Panel B). Table 3 presents results from exploratory multilevel moderated mediation models.

**Table 3:** Direct and indirect effects of group on recall differences between JOL groups from multilevel moderated mediation models for Experiments 1 and 2

	Direct effect			Indirect effect by learning strategy		
	*b*	95% CI	*p*	*b*	95% CI	*p*
*Experiment 1: Mediation by mental imagery*						
Related pairs	–0.015	[–0.067, 0.036]	.554	–0.016	[–0.036, 0.003]	.122
Unrelated pairs	**0.078**	**[0.020, 0.137]**	**.010**	**0.162**	**[0.125, 0.201]**	**<.001**
*Experiment 1: Joint mediation by mental imagery and no strategy*						
Related pairs	–0.002	[–0.055, 0.051]	.930			
Mental imagery				–0.012	[–0.028, 0.002]	.123
No strategy				–0.012	[–0.029, 0.005]	.163
Unrelated pairs	0.042	[–0.019, 0.103]	.180			
Mental imagery				**0.124**	**[0.090, 0.158]**	**<.001**
No strategy				**0.075**	**[0.048, 0.106]**	**<.001**
*Experiment 2: Mediation by no strategy*						
Related pairs	–0.003	[–0.073, 0.067]	.934	–0.030	[–0.060, 0.001]	.053
Unrelated pairs	**0.079**	**[0.007, 0.151]**	**.033**	**0.054**	**[0.016, 0.093]**	**.007**
*Experiment 2: Joint mediation by no strategy and mental imagery*						
Related pairs	–0.001	[–0.073, 0.071]	.974			
No strategy				–0.026	[–0.054, 0.001]	.054
Mental imagery				–0.006	[–0.019, 0.007]	.378
Unrelated pairs	0.054	[–0.013, 0.121]	.116			
No strategy				**0.046**	**[0.014, 0.081]**	**.007**
Mental imagery				**0.032**	**[0.014, 0.050]**	**<.001**

Confidence intervals represent Markov-chain-Monte-Carlo confidence intervals and were computed using the *bruceR* package (Bao, 2023). All variables in the moderated mediation models were dummy-coded (judgment group: 0 = JOL, 1 = MI/LS-JOL; cued-recall performance: 0 = not recalled, 1 = recalled; Relatedness: 0 = unrelated, 1 = related; Learning strategy: 0 = learning strategy not used, 1 = learning strategy used). Bold values are significant at *p* < .05

#### Experiment 1

An ANOVA (Table 2c) revealed significant effects of relatedness and judgment group, and a significant interaction. The main effects showed that recall was higher for related than for unrelated pairs and higher for the MI-JOL than for the JOL group (see Supplementary Materials). The interaction revealed that the effect of judgment group differed by relatedness. The JOL group exhibited positive reactivity for related pairs, *t*(127) = 2.46, *p* = .015,* d* = 0.43, and negative reactivity for unrelated pairs, *t*(127) = 4.56, *p* < .001, *d* = 0.80, whereas the MI-JOL group showed no reactivity, related pairs: *t*(126) = 1.66, *p* = .100,* d* = 0.29; unrelated pairs: *t*(126) = 0.94, *p* = .349,* d* = 0.17. Recall for unrelated pairs was higher in the MI-JOL than the JOL group, *t*(127) = 6.52, *p* < .001, *d* = 1.15.

Mediation analyses indicated that learning strategy use mediated differences between the JOL and MI-JOL groups in recall for unrelated but not for related pairs. Recall differences for unrelated pairs were partially mediated by mental imagery use, *b* = 0.162, *p* < .001, and fully mediated by mental imagery use combined with no strategy use, *b* = 0.124, *p* < .001. In contrast, recall differences for related pairs were not mediated by mental imagery or no strategy, *p*s $\ge$ .123.

#### Experiment 2

An ANOVA (Table 2c) revealed a significant effect of relatedness and a significant interaction, indicating higher recall was for related than for unrelated pairs and that the effect of judgment group differed by relatedness. The JOL group exhibited positive reactivity for related pairs, *t*(131) = 2.18, *p* = .031,* d* = 0.38, and negative reactivity for unrelated pairs, *t*(131) = 2.35, *p* = .020, *d* = 0.41, whereas the LS-JOL group showed no reactivity, related pairs: *t*(132) = 1.35, *p* = .180,* d* = 0.23; unrelated pairs: *t*(132) = 0.55, *p* = .585,* d* = 0.09. Recall for unrelated pairs was higher in the LS-JOL than in the JOL group, *t*(131) = 3.14, *p* = .002, *d* = 0.54.

Mediation analyses indicated that learning strategy use mediated differences between the JOL and LS-JOL groups in recall for unrelated but not for related pairs. Recall differences for unrelated pairs were partially mediated by no strategy use, *b* = 0.054, *p* < .001, and fully mediated by no strategy use combined with mental imagery use, *b* = 0.046, *p* = .007. In contrast, recall differences for related pairs were not mediated by no strategy or mental imagery, *p*s $\ge$ .053.

## General discussion

The present research examined whether instructing learning strategy use for unrelated pairs reduces negative JOL reactivity for these pairs. It thus aimed to provide further evidence for a contribution of learning strategies to negative reactivity. Two experiments showed that instructing JOL groups to use mental imagery (Experiment 1) or any learning strategy (Experiment 2) for unrelated pairs eliminated negative reactivity compared with standard JOL groups. These results provide converging evidence for previous mediation-based findings that changes in learning strategy use contribute to negative reactivity for unrelated pairs.

The present study fully replicated prior work by Ingendahl and Undorf (2025) revealing a correlative relationship between negative reactivity for unrelated pairs and reduced use of mental imagery and increased use of no strategy. The present results also converge with findings by Witherby and colleagues (2023) showing that mental-imagery instructions improve memory for unrelated pairs in JOL groups. Our findings thus add to accumulating research on the contribution of learning strategies to JOL reactivity—at least for negative effects of JOLs.

Alternatively, learning strategy instructions might have reduced negative reactivity by increasing depth of processing rather than because learning strategy use itself is causally related to negative reactivity.^4^ Incongruent with this possibility, both the present experiments and those by Ingendahl and Undorf (2025) consistently found that learning strategy use mediated negative reactivity, implying that learning strategies themselves contribute to negative reactivity. Of course, changes in learning strategy use for unrelated pairs might reduce depth of processing, which in turn leads to negative reactivity. That is, a lack of processing depth may explain negative reactivity on a higher level. Examining the interplay between learning strategy use, processing depth, and negative reactivity is therefore an important avenue for future research.

Considering established JOL reactivity theories, our results align with the dual-task and the changed-goal account (both Mitchum et al., 2016). Specifically, the finding that instructed strategy use eliminated negative reactivity is consistent with the dual-task account if dual-task costs of providing JOLs during studying increase spontaneous tendencies not to use strategies. If strategy instructions for unrelated pairs emphasize the importance of mastering these pairs and thereby counteract motivational tendencies to focus on related pairs when providing JOLs, our findings align with the changed-goal account. At the same time, neither account fully explains the present results: The dual-task account is inconsistent with positive reactivity for related pairs, and the changed-goal account cannot explain why learning strategies contribute to negative reactivity only (Ingendahl & Undorf, 2025; Rivers et al., 2021). The present findings are likewise inconsistent with the cue-strengthening account (Soderstrom et al., 2015), which attributes positive reactivity to strengthened processing of cues for JOLs, as it only accounts for positive reactivity for related pairs and for strategy differences that may be associated with them. Together with previous experimental (e.g., Chang & Brainerd, 2022; Janes et al., 2018; Myers et al., 2020; for a review, see Ingendahl et al., 2025) and meta-analytical evidence (Ingendahl et al., 2025), this study shows that new and refined theoretical accounts are needed to explain the complex processes and factors contributing to positive and negative reactivity (also see Myers et al., 2024). It may be promising to consider negative reactivity as arising from both motivational changes and limited cognitive resources, which are particularly pronounced when situational or individual factors discourage mastery goals or impose heavy demands on learners (e.g., through brief experimenter-paced study durations).

The present study manipulated learning strategy use only in JOL groups and only for unrelated pairs. Although this design allowed us to test the specific contribution of learning strategies to negative reactivity in unrelated pairs, it also entails limitations. First, our study provides relatively few insights into what drives positive reactivity. Analyses of learning strategy use in the standard JOL and no-JOL groups (see Supplementary Materials) replicate Ingendahl and Undorf (2025) in showing that the frequency of learning strategy use contributes minimally to positive reactivity (also see Rivers et al., 2021). It is still possible, however, that qualitative rather than quantitative changes in learning strategies related to deeper, more elaborative processing of pre-existing associative relations contribute to positive reactivity for related pairs (see cue-strengthening account and Ingendahl & Undorf, 2025). Second, results revealed that our learning strategy instructions targeted at unrelated pairs attenuated positive reactivity for related pairs. One possible explanation is that emphasizing unrelated pairs led to less learning strategy use for related pairs. Consistent with this, the JOL group with mental-imagery instructions used mental imagery less frequently for related pairs than the no-JOL group in Experiment 1. The minor role of learning strategies in positive reactivity (see above) suggests that strategy instructions for unrelated pairs did not influence positive reactivity through changes in learning strategy use. Instead, the mechanisms underlying positive JOL effects appear to be shaped by motivational factors such as prioritization and learning goals.

In conclusion, this study demonstrates that negative reactivity for unrelated pairs is eliminated when JOL-group participants are instructed to study unrelated pairs using mental imagery or another learning strategy. These findings provide converging evidence that learning strategies contribute to detrimental effects of making JOLs on memory performance.

## Supplementary Information

Below is the link to the electronic supplementary material.

Supplementary file1 (PDF 380 KB)

## Data Availability

All data and materials of the reported experiments are available at the Open Science Framework and can be accessed online (osf.io/3pnk9).

## Footnotes

1 A comprehensive review of research on learning strategies and JOL reactivity can be found in Ingendahl and Undorf (2025).

2 Additional power analyses are reported in the Supplementary Materials.

3 We exploratively examined participants’ scores in the Vividness of Visual Imagery Questionnaire. Three participants had scores considered as an indication of no visual imagery (Beran et al., 2023) and none of them were randomly assigned to the MI-JOL group. More information on the Vividness of Visual Imagery Questionnaire can be found in the Supplementary Materials .

4 We thank an anonymous reviewer for this suggestion.

## Code availability

All analytical code underlying the reported results is available at the Open Science Framework and can be accessed online (osf.io/3pnk9).
