# Supplementary material for: Instructed learning strategy use eliminates negative reactivity of immediate judgments of learning
Source: Psychon Bull Rev. 2026 Jan 14;33(1):47. doi: 10.3758/s13423-025-02844-w (PMC12804242; doi:10.3758/s13423-025-02844-w)
Supplement: Supplementary file 1 — Supplementary file1 (PDF 380 KB) [file 13423_2025_2844_MOESM1_ESM.pdf]

Supplementary Materials

Experiment 1

Vividness of Visual Imagery Questionnaire

Descriptive statistics for the translated version of the Vividness of Visual Imagery Questionnaire (Marks, 1973; translated was the version reported in Beran et al., 2023) by group are presented in Table S1.

Table S1

Descriptive Statistics of the Translated Version of the Vividness of Visual Imagery Questionnaire in Experiment 1

Judgment group	*M*	*SD*	*Min*	*Max*
JOL (*n* = 65)	53.28	11.62	16	80
MI-JOL (*n* = 64)	54.70	11.16	32	77
No JOL (*n* = 64)	49.67	12.57	16	78

Note. The questionnaire was taken from Beran and colleagues (2023) and translated into German. The questionnaire consists of 16 items which each ask participants to rate the vividness of their mental image belonging to one of four scenarios on a 5-point scale from 1 (“no image, you just *know* about the object “) to 5 (“perfectly clear and vivid as real seeing”). The sum score of the questionnaires ranges from 16 to 80, with higher values indicating higher vividness of visual imagery.

Effect of Counterbalancing Font-Color Assignment to Related and Unrelated Pairs

JOLs. We conducted an exploratory 2 (relatedness: related, unrelated) × 2 (judgment group: JOL, MI-JOL) × 2 (font color of unrelated pairs: green, purple) mixed ANOVA on JOLs with relatedness as within-subjects factor and judgment group and font color of unrelated pairs manipulated between participants. No main effects or interactions including font color were significant: Font color: $F(1, 125) = 0.35$, $p = .555$, $\eta_p^2 < .01$; Font color × Relatedness: F(1,

125) = 0.05, p = .815, η_p^2 < .01; Font color × Judgment group: F(1, 125) = 2.77, p = .098, η_p^2 = .02; Font color × Relatedness × Judgment group: F(1, 125) = 1.13, p = .290, η_p^2 = .01.

Mental Imagery Use. We conducted an exploratory 2 (relatedness: related, unrelated) × 3 (judgment group: JOL, MI-JOL, no JOL) × 2 (font color of unrelated pairs: green, purple) mixed ANOVA on the frequency of reporting mental imagery as a learning strategy with relatedness as within-subjects factor and judgment group and font color of unrelated pairs manipulated between participants. No main effects or interactions including font color were significant: Font color: F(1, 187) = 0.94, p = .334, η_p^2 = .01; Font color × Relatedness: F(1, 187) = 0.62, p = .433, η_p^2 < .01; Font color × Judgment group: F(2, 187) = 0.95, p = .387, η_p^2 = .01; Font color × Relatedness × Judgment group: F(2, 187) = 1.06, p = .349, η_p^2 = .01.

Cued Recall. We conducted an exploratory 2 (relatedness: related, unrelated) × 3 (judgment group: JOL, MI-JOL, no JOL) × 2 (font color of unrelated pairs: green, purple) mixed ANOVA on cued recall with relatedness as within-subjects factor and judgment group and font color of unrelated pairs manipulated between participants. No main effects or interactions including font color were significant: Font color: F(1, 187) = 1.48, p = .225, η_p^2 = .01; Font color × Relatedness: F(1, 187) = 0.01, p = .914, η_p^2 < .01; Font color × Judgment group: F(2, 187) = 1.37, p = .257, η_p^2 = .01; Font color × Relatedness × Judgment group: F(2, 187) = 0.30, p = .739, η_p^2 < .01.

Supplementary Analyses to the Main Analyses Reported in the Manuscript

Mental Imagery Use. The mixed ANOVA on mental imagery use reported in the manuscript's results section (see Table 2b) revealed a main effect of judgment group. We conducted exploratory two-sample t tests to assess the nature of this main effect. Results revealed that the main effect of judgment group was due to higher reported mental imagery use in the MI-JOL group, t(127) = 8.56, p < .001, d = 1.51, and the no-JOL group, t(127) =

4.06, $p < .001$, $d = 0.72$, compared to the JOL group, with the MI-JOL and the no-JOL groups not differing from each other, $t(126) = 1.98$, $p = .050$, $d = 0.35$.

Cued Recall. The mixed ANOVA on cued recall reported in the Results section in the manuscript (see Table 2c) revealed a main effect of judgment group. We conducted exploratory two-sample *t* tests to assess the nature of this main effect. Results revealed that the main effect of judgment group was due to higher recall in the MI-JOL group than in the JOL group, $t(127) = 4.04$, $p < .001$, $d = 0.71$, with no other groups differing from each other, MI-JOL vs. no JOL: $t(126) = 1.35$, $p = .181$, $d = 0.24$; JOL vs. no JOL: $t(127) = 1.82$, $p = .071$, $d = 0.32$. The ANOVA also revealed a significant interaction between relatedness and judgment group that was characterized by positive and negative reactivity for the JOL group but no reactivity for the MI-JOL group. To follow-up this interaction, we conducted preregistered paired *t* tests on the effect of relatedness in each judgment group: The effect of relatedness was significant in all groups but larger in the JOL group, $t(64) = 27.07$, $p < .001$, $d = 3.34$, than in the MI-JOL group, $t(63) = 12.68$, $p < .001$, $d = 1.57$, and the no-JOL group, $t(63) = 12.36$, $p < .001$, $d = 1.11$. This increased relatedness effect in the JOL group is considered an indication of JOL reactivity for related and unrelated word pairs (Janes et al., 2018; Undorf et al., 2024).

Differences in Learning Strategy Use Between the JOL and No-JOL groups

The frequency of reported learning strategy use by relatedness and judgment group is presented in Table S2. The following preregistered analyses focus on differences in self-reported use of learning strategies between the JOL and no-JOL groups, similar to the analyses reported by Ingendahl and Undorf (2025). A significant chi-squared test on all trials except those in which participants responded “I don’t know” revealed that learning strategy use differed between the JOL and no-JOL groups, $\chi^2(6) = 238.69$, $p < .001$, $\omega = 0.22$. An examination of the standardized residuals of the chi-squared test showed that residuals for making connections, mental imagery, no strategy, other strategy, retrieval practice, and

sentence generation were significantly larger than the z value considered as a cut-off value for influential values (i.e., 2.69, corresponding to a significance level of .05 corrected by the number of cells).

Table S2

Relative Frequency of Reported Item-Based Learning Strategies in Experiment 1

Experiment and condition	Reported learning strategy							
	Mental imagery	Making connections	Sentence generation	Rote repetition	Retrieval practice	Other strategy	No strategy	Do not know
JOL group								
Related word pairs	.14	.73	.02	.05	.01	.00	.03	.02
Unrelated word pairs	.15	.07	.04	.15	.08	.00	.36	.15
MI-JOL group								
Related word pairs	.09	.69	.01	.03	.05	.02	.07	.02
Unrelated word pairs	.66	.04	.03	.04	.04	.01	.11	.09
No-JOL group								
Related word pairs	.22	.58	.02	.05	.02	.01	.06	.02
Unrelated word pairs	.38	.10	.07	.14	.04	.01	.16	.10

Note. Sums per row may deviate from 1 due to rounding.

Mixed ANOVAs and t Tests on Frequency of Reporting Learning Strategies. As follow-up analyses, we performed separate Relatedness × Judgment group ANOVAs for each of these learning strategies. Full results of the mixed ANOVAs are presented in Table S3 and results of follow-up two-sample *t* tests on differences between the JOL and no-JOL groups are presented in Table S4. The results of the ANOVAs and *t* tests revealed that the JOL group reported to have used making connections more frequently and mental imagery less frequently for studying related word pairs than the no-JOL group. For unrelated word pairs, the results indicated that the JOL group reported to have used mental imagery less frequently but no strategy and retrieval practice more frequently than the no-JOL group. Further, the no-JOL group overall reported to have used another learning strategy more frequently than the JOL group. The JOL and no-JOL group did not differ significantly in the frequency of reporting sentence generation as a learning strategy.

Table S3

Results of the 2 (Relatedness: Related, Unrelated) × 2 (Judgment Group: JOL, No JOL) Mixed ANOVAs on the Frequency of Reporting Learning Strategies in Experiment 1

Learning strategy	Effect	F	df	p	η_p^2
Making connections					
	Relatedness	414.38	1, 127	< .001	.77
	**Judgment group**	**4.04**	**1, 127**	**.047**	**.03**
	**Relatedness × Judgment group**	**11.36**	**1, 127**	**< .001**	**.08**
Mental imagery					
	Relatedness	11.34	1, 127	< .001	.12
	**Judgment group**	**16.50**	**1, 127**	**.001**	**.08**
	**Relatedness × Judgment group**	**7.72**	**1, 127**	**.006**	**.06**
No strategy					
	Relatedness	81.30	1, 127	< .001	.39
	**Judgment group**	**7.27**	**1, 127**	**.008**	**.05**
	**Relatedness × Judgment group**	**24.20**	**1, 127**	**< .001**	**.16**
Other strategy					
	Relatedness	0.30	1, 127	.587	< .01

Learning strategy	Effect	*F*	*df*	*p*	η_p^2
	**Judgment group**	**6.49**	**1, 127**	**.012**	**.05**
	Relatedness × Judgment group	1.22	**1, 127**	.272	.01
Retrieval practice					
	Relatedness	20.33	1, 127	< .001	.14
	Judgment group	1.55	1, 127	.215	.01
	**Relatedness × Judgment group**	**8.07**	**1, 127**	**.005**	**.06**
Sentence Generation					
	Relatedness	8.66	1, 127	.004	.06
	Judgment group	1.32	1, 127	.252	.01
	Relatedness × Judgment group	1.33	1, 127	.252	.01

Note. We only examine learning strategies for which the standardized residuals for the chi-squared test indicated an influential contribution. Bold values indicate significant effects including Judgment group at $p < .05$.

Table S4

Results of Two-Sample *t* Tests on the Frequency of Reporting Learning Strategies in Experiment 1

Learning strategy	Word-pair relatedness	*t*	*df*	*p*	*d*
Making connections					
	**Related**	**2.85**	**127**	**.005**	**0.50**
	Unrelated	1.65	127	.102	0.29
Mental imagery					
	**Related**	**1.99**	**127**	**.049**	**0.35**
	**Unrelated**	**4.65**	**127**	**< .001**	**0.81**
No strategy					
	Related	1.17	127	.245	0.21
	**Unrelated**	**4.06**	**127**	**< .001**	**0.71**
Retrieval practice					
	Related	1.77	127	.080	0.31
	**Unrelated**	**2.05**	**127**	**.042**	**0.36**

Note. We only examine learning strategies for which the interaction effect among relatedness and judgment group was significant. Bold values indicate significant differences between the JOL and no-JOL groups at $p < .05$.

Multilevel Moderated Mediation Model. For the learning strategies that differed between the JOL and no-JOL groups, we ran multilevel moderated mediation models testing whether using the respective learning strategy mediated the effect of judgment group on cued-recall performance. To account for differences in JOL reactivity and learning strategy use between related and unrelated word pairs, each model included word-pair relatedness as a moderator of the direct effect of judgment group on cued-recall performance and learning strategy use. In a first step, we entered each learning strategy in a separate model and then, in a second step, combined all significant mediators of JOL reactivity in a joint model. We performed each multilevel moderated mediation model with the highest converging random effect structure. Results of all mediation models are presented in Table S5. Positive reactivity for related word pairs was mediated by making connections and other strategy (for similar findings, see Experiment 1b by Ingendahl & Undorf, 2025). Negative reactivity for unrelated word pairs was mediated by mental imagery and no strategy, replicating Ingendahl and Undorf (2025, Experiments 1a, 1b, 2a, and 2b). Retrieval practice did not mediate positive or negative reactivity.

Table S5

Direct and Indirect Effects of the Multilevel Moderated Mediation Models in Experiment 1

Word-pair types and learning strategy	Direct effect			Indirect effect by learning strategy		
	b	95% CI	*p*	*b*	95% CI	*p*
Model 1: Mediation by making connections						
Related pairs	0.06	[–0.003, 0.118]	.066	**0.029**	**[0.009, 0.051]**	**.008**
Unrelated pairs	**–0.186**	**[–0.269, –0.103]**	**< .001**	–0.006	[–0.015, 0.001]	.123
Model 2: Mediation by mental imagery						
Related pairs	**0.090**	**[0.030, 0.151]**	**.004**	–0.022	[–0.045, –0.001]	.055
Unrelated pairs	**–0.139**	**[–0.213, –0.065]**	**< .001**	**–0.059**	**[–0.090, –0.032]**	**< .001**
Model 3: Mediation by no strategy						
Related pairs	**0.062**	**[0.002, 0.123]**	**.046**	0.012	[–0.009, 0.034]	.249
Unrelated pairs	**–0.114**	**[–0.195, –0.034]**	**.006**	**–0.082**	**[–0.125, –0.040]**	**< .001**
Model 4: Mediation by other strategy						
Related pairs	**0.078**	**[0.019, 0.137]**	**.011**	**–0.003**	**[–0.006, –0.001]**	**.017**
Unrelated pairs	**–0.195**	**[–0.280, –0.111]**	**< .001**	–0.002	[–0.005, 0.001]	.222
Model 5: Mediation by retrieval practice						
Related pairs	**0.079**	**[0.021, 0.137]**	**.009**	0.001	[–0.000, 0.004]	.237
Unrelated pairs	**–0.180**	**[–0.259, –0.100]**	**< .001**	–0.004	[–0.012, 0.001]	.211
Model 6: Joint mediation by making connections, mental imagery, no strategy, and other strategy						
Related pairs	0.053	[–0.011, 0.116]	.105			
Making connections				**0.051**	**[0.030, 0.073]**	**<.001**
Mental imagery				**–0.032**	**[–0.061, –0.001]**	**.036**
No strategy				0.007	[–0.009, 0.023]	.370
Other strategy				**–0.005**	**[–0.009, –0.001]**	**.008**
Unrelated pairs	–0.053	[–0.120, 0.015]	.129			
Making connections				–0.011	[–0.032, 0.010]	.305
Mental imagery				**–0.084**	**[–0.116, –0.054]**	**< .001**
No strategy				**–0.047**	**[–0.064, –0.029]**	**< .001**

Word-pair types and learning strategy	Direct effect			Indirect effect by learning strategy		
	b	95% CI	*p*	*b*	95% CI	*p*
Other strategy				–0.002	[–0.006, 0.001]	.207

Note. Confidence intervals represent Markov-chain-Monte-Carlo confidence intervals and were computed using the *bruceR* package (Bao, 2023). All variables in the moderated mediation models were dummy-coded (Judgment group: 0 = no JOL, 1 = JOL; cued-recall performance: 0 = not recalled, 1 = recalled; Relatedness: 0 = unrelated, 1 = related; Learning strategy: 0 = learning strategy not used, 1 = learning strategy used). Bold values are significant at $p < .05$.

Experiment 2

Effect of Counterbalancing List Assignment

JOLs. We conducted an exploratory 2 (relatedness: related, unrelated) × 2 (judgment group: JOL, LS-JOL) × 2 (list: A, B) mixed ANOVA on JOLs with relatedness as within-subjects factor and judgment group and list manipulated between participants. A main effect of list indicated that JOLs for List A were higher than for List B, $F(1, 129) = 5.78$, $p = .018$, $\eta_p^2 = .04$, $M_A = 53.32$, $SD_A = 25.65$, $M_B = 48.03$, $SD_B = 25.28$. Because no interactions including list were significant, we did not perform any follow-up analyses separated by list: List × Relatedness: $F(1, 129) = 1.04$, $p = .310$, $\eta_p^2 = .01$; List × Judgment group: $F(1, 129) = 0.05$, $p = .815$, $\eta_p^2 < .01$; List × Relatedness × Judgment group: $F(1, 129) = 0.94$, $p = .333$, $\eta_p^2 = .01$.

No Strategy Use. We conducted an exploratory 2 (relatedness: related, unrelated) × 3 (judgment group: JOL, LS-JOL, no JOL) × 2 (list: A, B) mixed ANOVA on the frequency of reporting no strategy as a learning strategy with relatedness as within-subjects factor and judgment group and list manipulated between participants. No main effects or interactions including list were significant: List: $F(1, 194) = 0.83$, $p = .364$, $\eta_p^2 < .01$; List × Relatedness: $F(1, 194) = 0.06$, $p = .804$, $\eta_p^2 < .01$; List × Judgment group: $F(2, 194) = 0.59$, $p = .556$, $\eta_p^2 = .01$; List × Relatedness × Judgment group: $F(2, 194) = 2.35$, $p = .099$, $\eta_p^2 = .02$.

Cued Recall. We conducted an exploratory 2 (relatedness: related, unrelated) × 3 (judgment group: JOL, LS-JOL, no JOL) × 2 (list: A, B) mixed ANOVA on cued recall with relatedness as within-subjects factor and judgment group and list manipulated between participants. A significant interaction between list and relatedness, $F(1, 194) = 8.75$, $p = .003$, $\eta_p^2 = .04$, indicated that the relatedness effect was smaller for List A, $t(98) = 15.28$, $p < .001$, $d = 1.29$, than for List B, $t(100) = 18.34$, $p < .001$, $d = 1.93$. Because no other effects including list were significant, we did not perform any follow-up analyses separated by list: List: $F(1,$

194) = 0.54, p = .465, η_p^2 < .01; List × Judgment group: F(2, 194) = 0.48, p = .621, η_p^2 = .01; List × Relatedness × Judgment group: F(2, 194) = 0.45, p = .640, η_p^2 = .01.

Effect of Counterbalancing Font-Color Assignment to Related and Unrelated Pairs

JOLs. We conducted an exploratory 2 (relatedness: related, unrelated) × 2 (judgment group: JOL, LS-JOL) × 2 (font color of unrelated pairs: green, purple) mixed ANOVA on JOLs with relatedness as within-subjects factor and judgment group and font color of unrelated pairs manipulated between participants. No main effects or interactions including font color were significant: Font color: F(1, 129) = 1.35, p = .247, η_p^2 = .01; Font color × Relatedness: F(1, 129) = 1.98, p = .161, η_p^2 = .02; Font color × Judgment group: F(1, 129) = 0.13, p = .719, η_p^2 < .01; Font color × Relatedness × Judgment group: F(1, 129) = 0.65, p = .420, η_p^2 = .01.

No Strategy Use. We conducted an exploratory 2 (relatedness: related, unrelated) × 3 (judgment group: JOL, LS-JOL, no JOL) × 2 (font color of unrelated pairs: green, purple) mixed ANOVA on the frequency of reporting no strategy as a learning strategy with relatedness as within-subjects factor and judgment group and font color of unrelated pairs manipulated between participants. No main effects or interactions including font color of unrelated pairs were significant: Font color: F(1, 194) = 0.44, p = .510, η_p^2 < .01; Font color × Relatedness, F(1, 194) = 0.03, p = .872, η_p^2 < .01; Font color × Judgment group: F(2, 194) = 2.21, p = .113, η_p^2 = .02; Font color × Relatedness × Judgment group: F(2, 194) = 1.11, p = .332, η_p^2 = .01.

Cued Recall. We conducted an exploratory 2 (relatedness: related, unrelated) × 3 (judgment group: JOL, LS-JOL, no JOL) × 2 (font color of unrelated pairs: green, purple) mixed ANOVA on cued recall with relatedness as within-subjects factor and judgment group and font color of unrelated pairs manipulated between participants. Because no main effects or interactions including font color were significant, we performed no follow-up analyses separated by list: Font color: F(1, 194) = 0.68, p = .409, η_p^2 < .01; Font color × Relatedness:

$F(1, 194) = 0.39$, $p = .536$, $\eta_p^2 < .01$; Font color × Judgment group: $F(2, 194) = 0.75$, $p = .474$, $\eta_p^2 = .01$; Font color × Relatedness × Judgment group: $F(2, 194) = 0.13$, $p = .875$, $\eta_p^2 < .01$.

Supplementary Analyses to the Main Analyses for Cued Recall Reported in the Manuscript

Cued Recall. The mixed ANOVA on cued recall reported in the Results section in the manuscript (see Table 2c) revealed a significant interaction between relatedness and judgment group that was characterized by positive and negative reactivity for the JOL group but no reactivity for the LS-JOL group. To follow-up this interaction, we conducted preregistered paired *t* tests on the effect of relatedness in each judgment group: The effect of relatedness was significant in all groups but larger in the JOL group, $t(65) = 17.48$, $p < .001$, $d = 2.20$, than in the LS-JOL group, $t(66) = 13.13$, $p < .001$, $d = 1.46$, and the no-JOL group, $t(66) = 12.51$, $p < .001$, $d = 1.17$.

Differences in Learning Strategy Use Between the JOL and No-JOL groups

The frequency of reported learning strategy use separate by relatedness and judgment group is presented in Table S6. The following preregistered analyses focus on differences in self-reported use of learning strategies between the JOL and no-JOL groups. A significant chi-squared test on all trials except those in which participants responded "I don't know" revealed that learning strategy use differed between the JOL and no-JOL groups, $\chi^2(6) = 97.92$, $p < .001$, $\omega = 0.14$. An examination of the standardized residuals of the chi-squared test showed that residuals for mental imagery, no strategy, retrieval practice, and sentence generation were significantly larger than the *z* value considered as a cut-off value for influential values.

Table S6

Relative Frequency of Reported Item-Based Learning Strategies in Experiment 2

Experiment and condition	Reported learning strategy							
	Mental imagery	Making connections	Sentence generation	Rote repetition	Retrieval practice	Other strategy	No strategy	Do not know
JOL group								
Related word pairs	.18	.60	.04	.04	.04	.00	.07	.04
Unrelated word pairs	.17	.11	.05	.09	.08	.01	.34	.15
LS-JOL group								
Related word pairs	.15	.58	.04	.04	.01	.00	.14	.02
Unrelated word pairs	.34	.11	.11	.07	.05	.03	.20	.09
No-JOL group								
Related word pairs	.17	.60	.05	.05	.01	.00	.08	.04
Unrelated word pairs	.29	.10	.08	.13	.04	.01	.22	.13

Note. Sums per row may deviate from 1 due to rounding.

Mixed ANOVAs and t Tests on Frequency of Reporting Learning Strategies. As follow-up analyses, we performed separate Relatedness × Judgment group ANOVAs for each of these learning strategies. Full results of the mixed ANOVAs are presented in Table S7 and results of follow-up two-sample *t* tests on differences between the JOL and no-JOL groups are presented in Table S8. The results of the ANOVAs and *t* tests revealed that the JOL group reported to have used mental imagery less frequently and no strategy more frequently for studying unrelated word pairs than the no-JOL group. Further, the no-JOL group reported to have used retrieval practice more frequently for related and unrelated word pairs than the JOL group. The JOL and no-JOL groups did not differ significantly in the frequency of reporting sentence generation as a learning strategy.

Table S7

Results of the 2 (Relatedness: Related, Unrelated) × 2 (Judgment Group: JOL, No JOL) Mixed ANOVAs on the Frequency of Reporting Learning Strategies in Experiment 2

Learning strategy	Effect	*F*	*df*	*p*	η_p^2
Mental imagery					
	Relatedness	4.55	1, 131	.035	.03
	Judgment group	2.44	1, 131	.121	.02
	**Relatedness × Judgment group**	**6.36**	**1, 131**	**.013**	**.05**
No strategy					
	Relatedness	86.02	1, 131	< .001	.40
	Judgment group	2.18	1, 131	.142	.02
	**Relatedness × Judgment group**	**8.25**	**1, 131**	**.005**	**.06**
Retrieval practice					
	Relatedness	11.51	1, 131	< .001	.08
	**Judgment group**	**4.65**	**1, 131**	**.033**	**.03**
	Relatedness × Judgment group	0.38	1, 131	.540	< .01
Sentence Generation					
	Relatedness	3.32	1, 131	.071	.03
	Judgment group	2.03	1, 131	.156	.02
	Relatedness × Judgment group	0.66	1, 131	.419	.01

Note. We only examine learning strategies for which the standardized residuals for the chi-squared test indicated an influential contribution. Bold values indicate significant effects including Judgment group at $p < .05$.

Table S8

Results of Two-Sample *t* Tests on the Frequency of Reporting Learning Strategies in Experiment 2

Learning strategy	Word-pair relatedness	*t*	*df*	*p*	*d*
Mental imagery					
	Related	0.36	131	.716	0.06
	**Unrelated**	**2.58**	**131**	**.011**	**0.45**
No strategy					
	Related	0.52	131	.607	0.09
	**Unrelated**	**2.37**	**131**	**.019**	**0.41**

Note. We only examine learning strategies for which the interaction effect among relatedness and judgment group was significant. Bold values indicate significant differences between the JOL and no-JOL groups at $p < .05$.

Multilevel Moderated Mediation Model. For the learning strategies that differed between the JOL and no-JOL groups, we ran multilevel moderated mediation models testing whether using the respective learning strategy mediated the effect of judgment group on cued-recall performance. To account for differences in JOL reactivity and learning strategy use between related and unrelated word pairs, each model included word-pair relatedness as a moderator of the direct effect of judgment group on cued-recall performance and learning strategy use (see Experiment 1). Results of all mediation models are presented in Table S9. The results of the mediation models were overall consistent with those reported by Ingendahl and Undorf (2025). Negative reactivity for unrelated word pairs was mediated by no strategy and mental imagery, whereas positive reactivity for related word pairs was not mediated by any learning strategy. Retrieval practice did not mediate positive or negative reactivity.

Table S9

Direct and Indirect Effects of the Multilevel Moderated Mediation Models in Experiment 2

Word-pair types and learning strategy	Direct effect			Indirect effect by learning strategy		
	b	95% CI	*p*	*b*	95% CI	*p*
Model 1: Mediation by mental imagery						
Related pairs	**0.074**	**[0.002, 0.145]**	**.045**	0.004	[–0.020, 0.028]	.713
Unrelated pairs	–0.070	[–0.148, 0.007]	.076	**–0.037**	**[–0.068, –0.009]**	**.015**
Model 2: Mediation by no strategy						
Related pairs	**0.089**	**[0.026, 0.153]**	**.007**	0.007	[–0.020, 0.033]	.609
Unrelated pairs	–0.010	[–0.069, 0.049]	.738	**–0.050**	**[–0.097, –0.005]**	**.030**
Model 3: Mediation by retrieval practice						
Related pairs	**0.080**	**[0.010, 0.150]**	**.027**	–0.002	[–0.007, 0.001]	.281
Unrelated pairs	**–0.096**	**[–0.183, –0.008]**	**.034**	–0.003	[–0.010, 0.001]	.303
Model 3: Mediation by mental imagery and no strategy						
Related pairs	**0.074**	**[0.009, 0.139]**	**.028**			
Mental imagery				0.003	[–0.012, 0.017]	.703
No strategy				0.006	[–0.021, 0.033]	.656
Unrelated pairs	–0.013	[–0.071, 0.046]	.674			
Mental imagery				**–0.024**	**[–0.041, –0.009]**	**.003**
No strategy				**–0.045**	**[–0.074, –0.016]**	**.003**

Note. Confidence intervals represent Markov-chain-Monte-Carlo confidence intervals and were computed using the *bruceR* package (Bao, 2023). All variables in the moderated mediation models were dummy-coded (Judgment group: 0 = no JOL, 1 = JOL; cued-recall performance: 0 = not recalled, 1 = recalled; Relatedness: 0 = unrelated, 1 = related; Learning strategy: 0 = learning strategy not used, 1 = learning strategy used). Bold values are significant at $p < .05$.

Exploratory Robustness Checks

Correction for Alpha-Inflation Due to Multiple Comparisons

Based on the suggestion of an anonymous reviewer, we applied Bonferroni-Holm corrections to all *t* tests conducted as part of the main analyses as a robustness check for the results in Experiments 1 and 2. An overview of the original and corrected *p* values is presented in Table S10. Results revealed that interpretations based on uncorrected and corrected *p* values were consistent, with only minor exceptions. With corrected *p* values, positive reactivity for related word pairs and negative reactivity for unrelated word pairs in Experiment 2's standard JOL group were only marginally significant ($p < .10$). Given that we used two-sided tests for both reactivity effects despite preregistering directional hypotheses, this does not provide evidence against positive and negative reactivity.

Table S10

Comparison of Original and Bonferroni-Holm Corrected *p* Values for *t* Tests Conducted in Experiments 1 and 2

Experiment and DV	Comparison	$p_{original}$	$p_{corrected}$
Experiment 1			
JOLs			
	Related pairs: JOL group ≠ MI-JOL group	.924	.924
	Unrelated pairs: JOL group ≠ MI-JOL group	< .001	< .001
Mental imagery			
	MI-JOL group ≠ JOL group	< .001	< .001
	No-JOL group ≠ JOL group	< .001	< .001
	Related pairs: MI-JOL group ≠ no-JOL group	< .001	.003
	**Related pairs: JOL group ≠ no-JOL group**	**.049**	**.097**
	Related pairs: MI-JOL group ≠ JOL group	.118	.118
	Unrelated pairs: MI-JOL group ≠ no-JOL group	< .001	< .001
	Unrelated pairs: JOL group ≠ no-JOL group	< .001	< .001
	Unrelated pairs: MI-JOL group ≠ JOL group	< .001	< .001

Experiment and DV	Comparison	$p_{original}$	$p_{corrected}$
Cued recall			
	MI-JOL group ≠ no-JOL group	.181	.362
	JOL group ≠ no-JOL group	.071	.284
	MI-JOL group ≠ JOL group	< .001	.001
	JOL group: Related pairs ≠ unrelated pairs	< .001	< .001
	MI-JOL group: Related pairs ≠ unrelated pairs	< .001	< .001
	No-JOL group: Related pairs ≠ unrelated pairs	< .001	< .001
	**Related pairs: JOL group ≠ no-JOL group**	**.015**	**.076***
	Unrelated pairs: JOL group ≠ no-JOL group	< .001	< .001
	Related pairs: MI-JOL group ≠ no-JOL group	.100	.300
	Unrelated pairs: MI-JOL group ≠ no-JOL group	.349	.362
	Unrelated pairs: MI-JOL group ≠ JOL group	< .001	< .001
Experiment 2			
JOLs			
	Related pairs: JOL group ≠ LS-JOL group	.750	.750
	Unrelated pairs: JOL group ≠ LS-JOL group	< .001	< .001
No strategy			
	Related pairs: LS-JOL group ≠ no-JOL group	.114	.343
	Related pairs: JOL group ≠ no-JOL group	.607	> .999
	Related pairs: LS-JOL group ≠ JOL group	.055	.220
	Unrelated pairs: LS-JOL group ≠ no-JOL group	.629	> .999
	**Unrelated pairs: JOL group ≠ no-JOL group**	**.019**	**.097**
	Unrelated pairs: LS-JOL group ≠ JOL group	.005	.028
Cued recall			
	JOL group: Related pairs ≠ unrelated pairs	< .001	< .001
	LS-JOL group: Related pairs ≠ unrelated pairs	< .001	< .001
	No-JOL group: Related pairs ≠ unrelated pairs	< .001	< .001
	**Related pairs: JOL group ≠ no-JOL group**	**.031**	**.092***
	**Unrelated pairs: JOL group ≠ no-JOL group**	**.020**	**.082***
	Related pairs: LS-JOL group ≠ no-JOL group	.180	.360
	Unrelated pairs: LS-JOL group ≠ no-JOL group	.585	.585
	Unrelated pairs: LS-JOL group ≠ JOL group	.002	.010

Note. $p_{original}$ refers to the original, uncorrected p value of the respective t test and $p_{corrected}$ refers to the Bonferroni-Holm corrected p value. Bonferroni-Holm corrections were

applied within each experiment and dependent variable. All original p values stemmed from two-sided t tests. Bold values indicate diverging interpretations based on the original and corrected p values.

* Would be considered significant at $p < .05$ when applying a one-sided t test for testing the preregistered directional psychological hypothesis.

Bayesian Analyses

As suggested during the review process, we additionally report exploratory Bayesian Analyses on preregistered ANOVAs in Table S11 and on preregistered t tests in Table S12. All Bayesian analyses were conducted using the *BayesFactor* package (Morey & Rouder, 2024) and were based on default priors. Overall, the Bayesian Analyses corroborate the frequentist analyses reported in the Results section by revealing mainly moderate to strong evidence for significant effects.

Table S11

Bayes Factors for Bayesian ANOVAs

Experiment and dependent variable	Effect	BF_{10}	Evidence
Experiment 1			
JOLs			
	Relatedness (H2)	6.65×10^{75}	Strong
	JOL Condition	7,999.24	Strong
	Relatedness × JOL Condition	5,591,241	Strong
Mental imagery			
	Relatedness	1.31×10^{15}	Strong
	Condition	1,212,386	Strong
	Relatedness × Condition (H3)	3.04×10^{17}	Strong

Experiment and dependent variable	Effect	BF_{10}	Evidence
Cued recall			
	Relatedness (H1)	4.31×10^{71}	Strong
	Condition	9.04	Moderate
	Relatedness × Condition (H4)	2.43×10^{13}	Strong
Experiment 2			
JOLs			
	Relatedness (H2)	3.86×10^{55}	Strong
	JOL Condition	2.67	Anecdotal
	Relatedness × JOL Condition	6.03	Moderate
No strategy			
	Relatedness	1.95×10^{9}	Strong
	Condition	0.11	Moderate*
	Relatedness × Condition (H3)	73.66	Strong
Cued recall			
	Relatedness (H1)	2.08×10^{58}	Strong
	Condition	0.18	Moderate*
	Relatedness × Condition (H4)	17,694.83	Strong

Note. Values in parentheses refer to tested hypothesis specified in the respective preregistration. BF_{10} refer to the following model comparisons: BF_{10}(Relatedness) = BF_{10}(Relatedness + Condition)/ BF_{10}(Condition); BF_{10}(Condition) = BF_{10}(Relatedness + Condition)/ BF_{10}(Relatedness); BF_{10}(Relatedness × Condition) = BF_{10}(Relatedness + Condition + Relatedness × Condition)/ BF_{10}(Relatedness + Condition). We interpret Bayes factors based on the following conventions: < 1/10 = strong evidence for H_0; 1/10–1/3 = moderate evidence for H_0; 1/3–1 = anecdotal evidence for H_0; 1–3 = anecdotal evidence for H_1; 3–10 = moderate evidence for H_1; >10 = strong evidence for H_1 (van Doorn et al., 2021).

* Indicates evidence in favor of the H0

Table S12

Bayes Factors for Bayesian *t* Tests

Experiment and dependent variable	Comparison	BF_{10}	Evidence
Experiment 1			
Mental imagery			
	Unrelated pairs: MI-JOL group ≠ JOL group (H3)	6.15×10^{17}	Strong
Cued recall			
	JOL group: Related ≠ unrelated (H4)	2.34×10^{33}	Strong
	MI-JOL group: Related ≠ unrelated (H4)	9.94×10^{15}	Strong
	No-JOL group: Related ≠ unrelated (H4)	3.21×10^{15}	Strong
	Related: JOL group ≠ no-JOL group (H4a)	2.83	Anecdotal
	Related: MI-JOL group ≠ no-JOL group (H4a)	0.65	Anecdotal*
	Unrelated: JOL group ≠ no-JOL group (H4b)	1469.52	Strong
	Unrelated: MI-JOL group ≠ no-JOL group (H4b)	0.28	Moderate*
	Unrelated: MI-JOL group ≠ JOL group (H5)	6,098,140	Strong
Experiment 2			
No strategy			
	Unrelated pairs: LS-JOL group ≠ JOL group (H3)	7.49	Moderate
Cued recall			
	JOL group: Related ≠ unrelated (H4)	1.20×10^{23}	Strong
	LS-JOL group: Related ≠ unrelated (H4)	1.11×10^{17}	Strong
	No-JOL group: Related ≠ unrelated (H4)	1.19×10^{16}	Strong
	Related: JOL group ≠ no-JOL group (H4a)	1.59	Anecdotal
	Related: LS-JOL group ≠ no-JOL group (H4a)	0.42	Moderate*
	Unrelated: JOL group ≠ no-JOL group (H4b)	2.22	Anecdotal
	Unrelated: LS-JOL group ≠ no-JOL group (H4b)	0.21	Moderate*
	Unrelated: LS-JOL group ≠ JOL group (H5)	14.95	Strong

Note. Values in parentheses refer to tested hypothesis specified in the respective preregistration. We interpret Bayes factors based on the following conventions: < 1/10 = strong evidence for H_0; 1/10–1/3 = moderate evidence for H_0; 1/3–1 = anecdotal evidence for H_0; 1–3 = anecdotal evidence for H_1; 3–10 = moderate evidence for H_1; >10 = strong evidence for H_1 (van Doorn et al., 2021).

* Indicates evidence in favor of the H0

Additional Power Analyses

ANOVAs on the Effect of Counterbalancing Color/List Assignment. We conducted exploratory power analyses using G*Power (Faul et al., 2007) for the conducted ANOVAs on the effect of counterbalancing color or list assignment to examine the sensitivity and post hoc power for the main effect and interactions involving counterbalancing factors based on the planned sample size of n = 64 participants per group. Sensitivity power analyses were based on planned sample sizes of N = 192 (for analyses on learning strategy use and cued-recall performance) and N = 128 (for analyses on JOLs), α = .05, and $(1 - \beta)$ = .80. Post hoc power analyses were based on an assumed moderate effect size of f = .25, α = .05, and planned sample sizes of N = 192 (for analyses on learning strategy use and cued-recall performance) and N = 128 (for analyses on JOLs). An overview of the sensitivity and post hoc power indicated by the exploratory power analyses is presented in Tables S13 and S14. Overall, sensitivity power analyses indicated that the experiments allowed for detecting small-to-moderate ($f \geq .10$) main or interaction effect of the counterbalancing factor with α = .05 and $(1 - \beta)$ = .80.

Table S13

Sensitivity Power Analyses for the ANOVAs on the Effect of Counterbalancing Font Color or List Assignment

Analysis and Assumptions	Effect	f
2 (Relatedness) × 2 (Condition) × 2 (Color/List) Mixed ANOVA on JOLs with $N = 128$, $\alpha = .05$, $(1 - \beta) = .80$		
	Color/List	.22
	Relatedness × Color/List	.12
	Condition × Color/List	.26
	Relatedness × Condition × Color/List	.15
2 (Relatedness) × 3 (Condition) × 2 (Color/List) Mixed ANOVA on learning strategies/recall with $N = 192$, $\alpha = .05$, $(1 - \beta) = .80$		
	Color/List	.18
	Relatedness × Color/List	.10
	Condition × Color/List	.23
	Relatedness × Condition × Color/List	.13

Note. All sensitivity analyses were conducted assuming a correlation among repeated measures of .50.

Table S14

Post Hoc Power Analyses for the ANOVAs on the Effect of Counterbalancing Font Color or List Assignment

Analysis and Assumptions	Effect	$1 - \beta$
2 (Relatedness) × 2 (Condition) × 2 (Color/List) Mixed ANOVA on JOLs with $N = 128$, $\alpha = .05$, $f = .25$		
	Color/List	.90
	Relatedness × Color/List	>.99

Analysis and Assumptions	Effect	1 – β
	Condition × Color/List	.78
	Relatedness × Condition × Color/List	.99
2 (Relatedness) × 3 (Condition) × 2 (Color/List) Mixed ANOVA on learning strategies/recall with $N = 192$, $\alpha = .05$, $f = .25$		
	Color/List	.98
	Relatedness × Color/List	>.99
	Condition × Color/List	.88
	Relatedness × Condition × Color/List	>.99

Note. All sensitivity analyses were conducted assuming a correlation among repeated measures of .50.

Multilevel Moderated Mediation Models. No straightforward power analysis is available for indirect effects in multilevel moderated mediation models. As a conservative approximation of the power for detecting the indirect effect of judgment group on cued-recall performance via learning strategy use for related and unrelated word pairs, we performed exploratory post hoc power analyses for the indirect effect at the participant level ignoring the multilevel structure. For simplicity, we limited the post hoc power analysis to the separate mediation effects of mental imagery and no strategy (i.e., the most theoretically relevant mediators) in Experiments 1 and 2. All post hoc power analyses were conducted using the software by Schoemann and colleagues (2017) and were based on the participant-level correlations between judgment group, learning strategy use (mental imagery/no strategy), and cued-recall performance separately for related and unrelated word pairs. Results indicated that the experiments had appropriate power to detect an indirect effect of judgment group on cued-recall performance mediated via learning strategy use for unrelated word pairs (Experiment 1:

$1 - \beta = .99/.97$; Experiment 2: $1 - \beta = .70/.66$), but lacked power for detecting the negligible effects for related word pairs (Experiment 1: $1 - \beta = .22/.12$; Experiment 2: $1 - \beta = .00/.07$).

References

Bao, H.-W.-S. (2023). *bruceR: Broadly Useful Convenient and Efficient R Functions* (Version 2023.9) [Computer software]. https://CRAN.R-project.org/package=bruceR

Beran, M. J., James, B. T., French, K., Haseltine, E. L., & Kleider-Offutt, H. M. (2023). Assessing aphantasia prevalence and the relation of self-reported imagery abilities and memory task performance. *Consciousness and Cognition*, *113*, 103548. https://doi.org/10.1016/j.concog.2023.103548

Faul, F., Erdfelder, E., Lang, A.-G., & Buchner, A. (2007). G*Power 3: A flexible statistical power analysis program for the social, behavioral, and biomedical sciences. *Behavior Research Methods*, *39*(2), 175–191. https://doi.org/10.3758/BF03193146

Ingendahl, F., & Undorf, M. (2025). Changes in learning strategies contribute to negative reactivity of immediate judgments of learning. *Journal of Experimental Psychology: Learning, Memory, and Cognition*. Advance online publication. https://doi.org/10.1037/xlm0001475

Janes, J. L., Rivers, M. L., & Dunlosky, J. (2018). The influence of making judgments of learning on memory performance: Positive, negative, or both? *Psychonomic Bulletin & Review*, *25*(6), 2356–2364. https://doi.org/10.3758/s13423-018-1463-4

Marks, D. F. (1973). VISUAL IMAGERY DIFFERENCES IN THE RECALL OF PICTURES. *British Journal of Psychology*, *64*(1), 17–24. https://doi.org/10.1111/j.2044-8295.1973.tb01322.x

Morey, R. D., & Rouder, J. N. (2024). *BayesFactor: Computation of Bayes Factors for Common Designs* (Version 0.9.12-4.7) [Computer software]. https://CRAN.R-project.org/package=BayesFactor

Schoemann, A. M., Boulton, A. J., & Short, S. D. (2017). Determining power and sample size for simple and complex mediation models. *Social Psychological and Personality Science*, *8*(4), 379–386. https://doi.org/10.1177/1948550617715068

Undorf, M., Ingendahl, F., & Halamish, V. (2024). Making judgments of learning either enhances or impairs memory: Evidence from 17 experiments with related and unrelated word pairs. *Collabra: Psychology*, *10*(1), 117108. https://doi.org/10.1525/collabra.117108

van Doorn, J., van den Bergh, D., Böhm, U., Dablander, F., Derks, K., Draws, T., Etz, A., Evans, N. J., Gronau, Q. F., Haaf, J. M., Hinne, M., Kucharský, Š., Ly, A., Marsman, M., Matzke, D., Gupta, A. R. K. N., Sarafoglou, A., Stefan, A., Voelkel, J. G., & Wagenmakers, E.-J. (2021). The JASP guidelines for conducting and reporting a Bayesian analysis. *Psychonomic Bulletin & Review*, *28*(3), 813–826. https://doi.org/10.3758/s13423-020-01798-5
